# Supplementary material for: Signal peptidase SpsB coordinates staphylococcal cell cycle, surface protein septal trafficking, and LTA synthesis
Source: mBio. 2025 Jan 24;16(3):e02673-24. doi: 10.1128/mbio.02673-24 (PMC11898559; doi:10.1128/mbio.02673-24)
Supplement: Supplemental Material — Figures S1 to S5; Tables S1 and S2. [file mbio.02673-24-s0001.docx]

**Supplemental Materials**
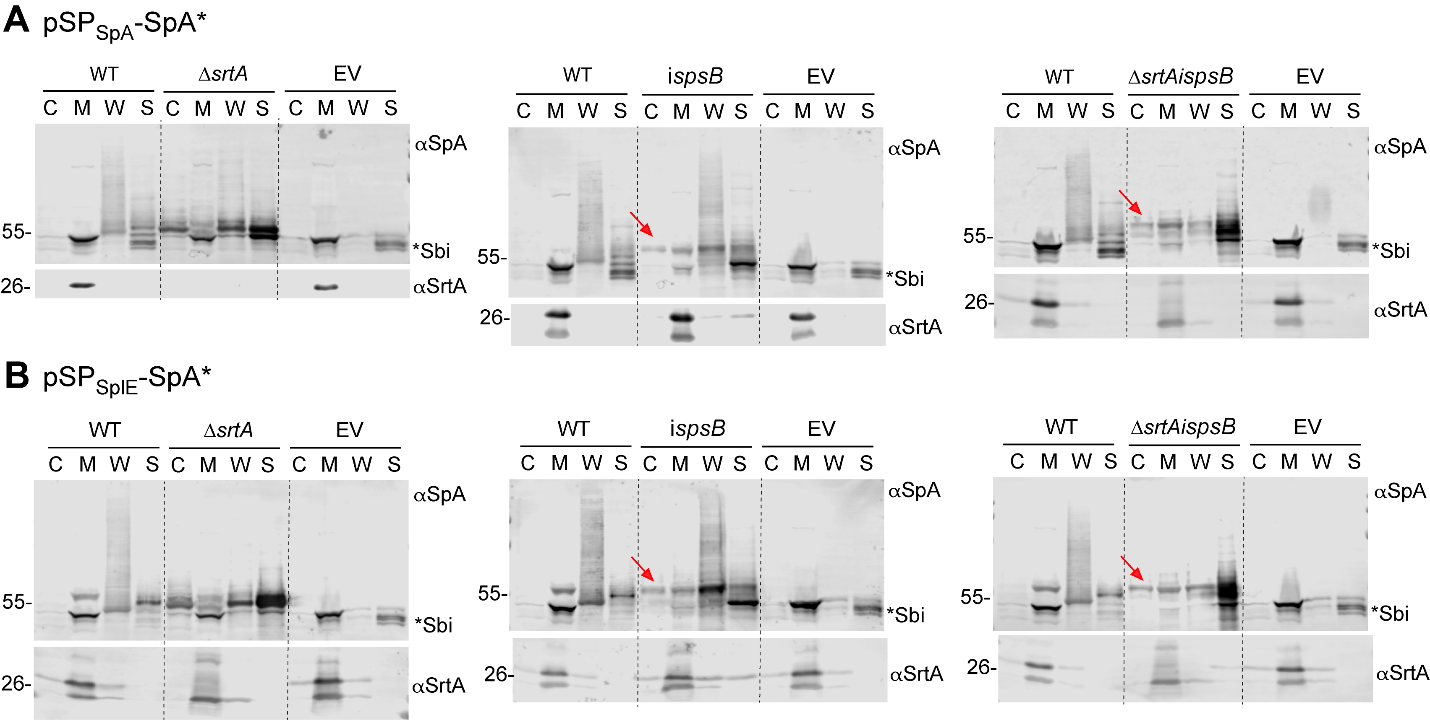


**Fig. S1.** Cell fractionation and immunoblot analysis SpA* with pKK30itet empty vector (EV) control. Bacterial cultures of SEJ1 WT, ∆*srtA*, *ispsB*, ∆*srtA/ispsB* expressing SpA* fused with SP_SpA_ or SP_SplE_ were fractionated to cytoplasm (C), cell membrane (M), cell wall (W), and the supernatant (S). All the strains were grown without IPTG to deplete *spsB* and with ATc to induce *spa** expression. The αSrtA blot is a loading and fractionation control. The red arrow indicates unprocessed SP-bearing precursors. The asterisk indicates non-specific Sbi bands. Numbers on the left indicate protein ladder in kDa.


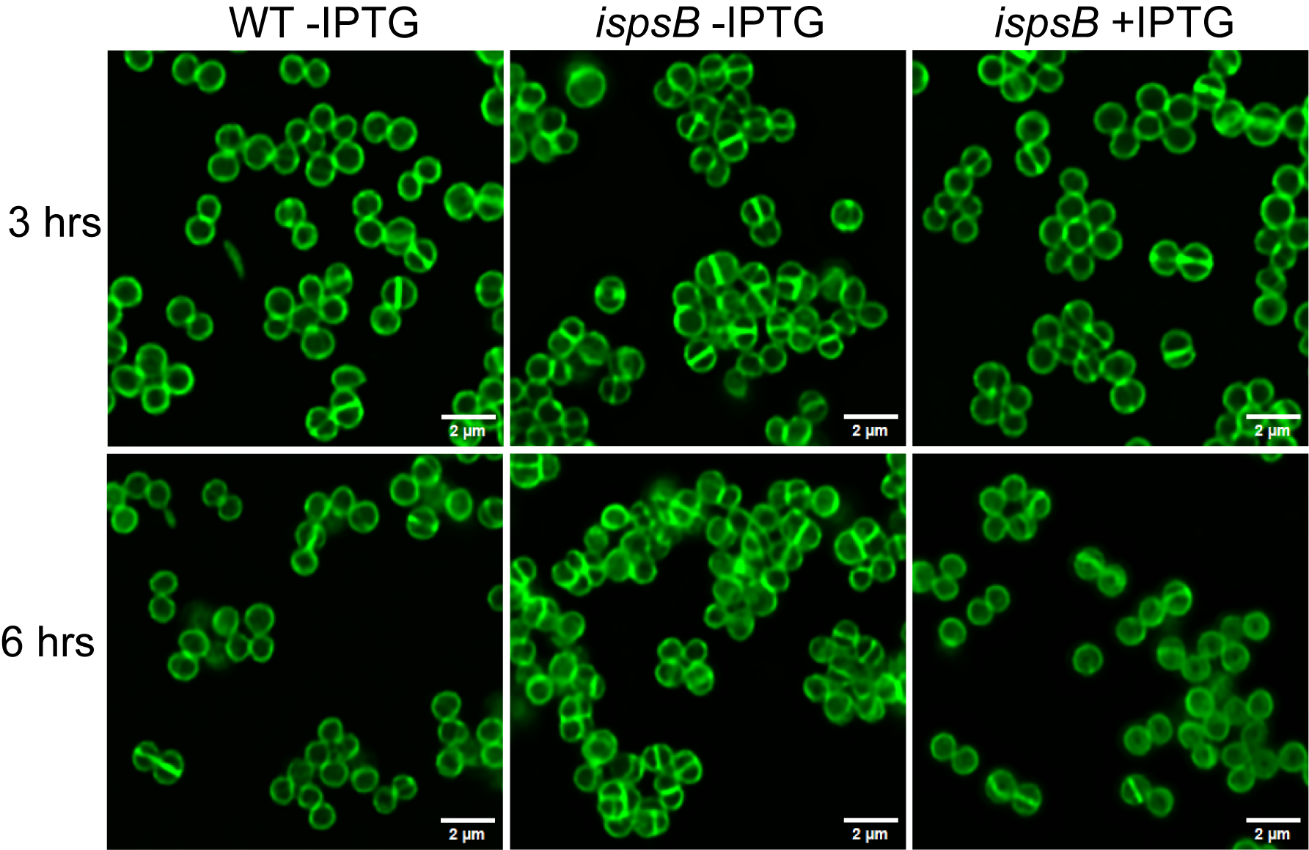


**Fig. S2.** Extended *spsB* depletion led to elevated cell cycle arrest. Staphylococcal cells were stained with Van-FL after 3- and 6-hours of *spsB* depletion.

**
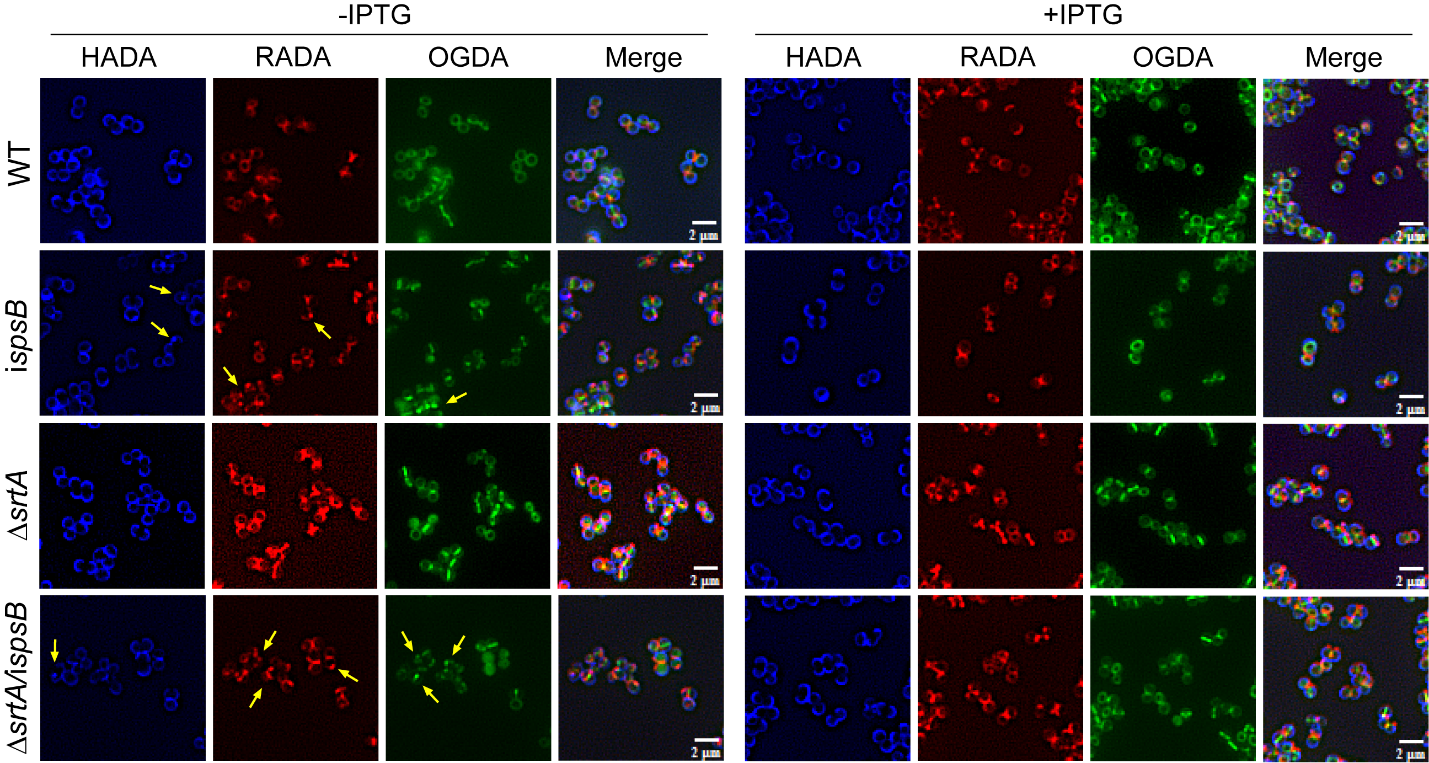
**

**Fig. S3.** Extended data figure of Fig. 5. Larger image crops showing defects of FDAA incorporation upon *spsB* depletion, indicated by yellow arrows.

**
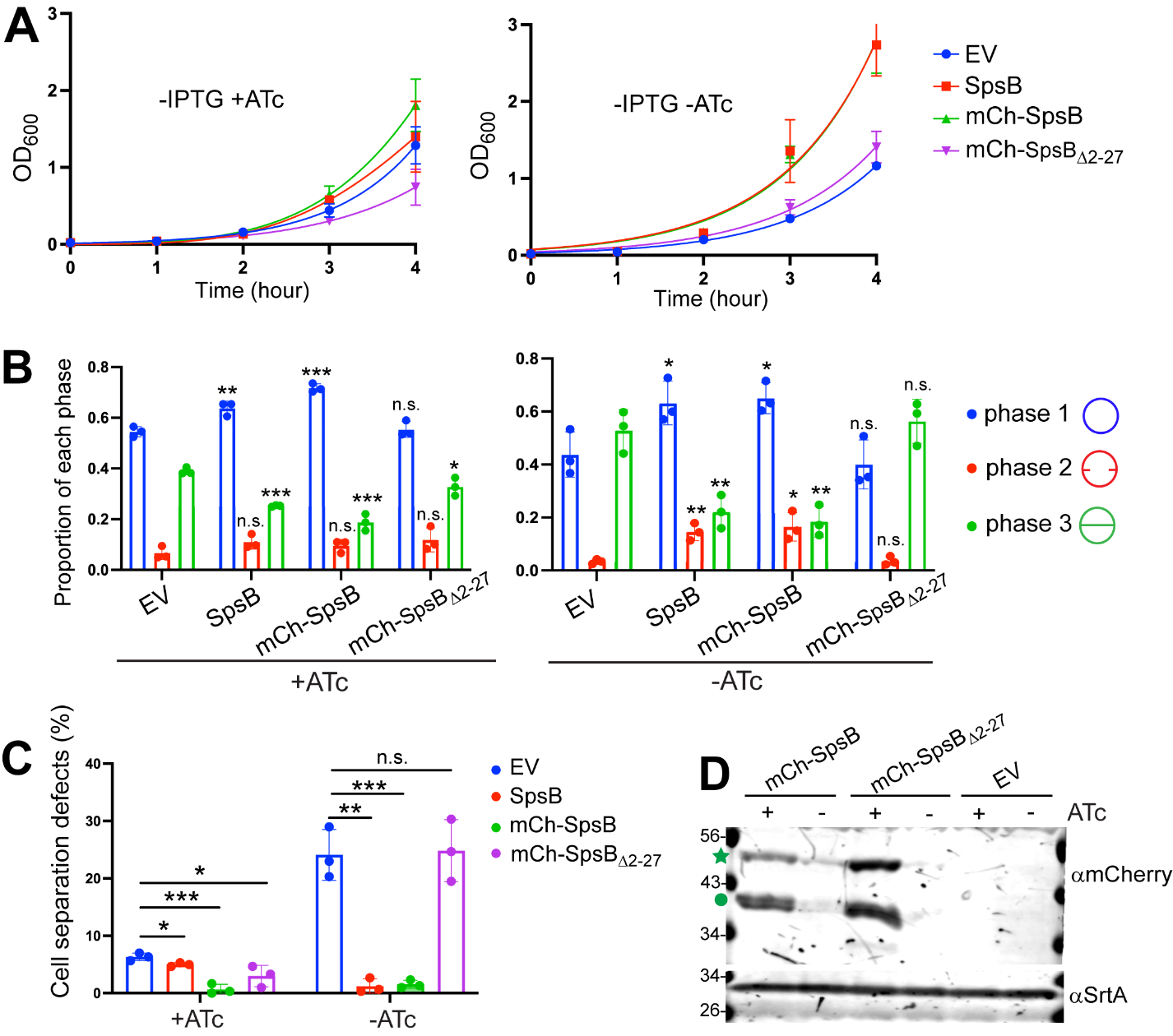
Fig. S4. The mCherry-SpsB fusion is functional. (A)** Growth curves of SEJ1*ispsB* expressing pKK30*itet* empty vector (EV), SpsB, mCherry-SpsB, mCherry-SpsB_Δ2-27_. Bacterial cultures were grown without IPTG to deplete *spsB* and with or without ATc to control the expression of the fusion proteins. **(B)** Quantification of cells from different stages of the cell cycle: with no septum (denoted as P1), a partial septum (denoted as P2), or a complete septum (denoted as P3). Asterisks on top of each sample indicate statistical analysis result between EV and the sample: *p <0.05; **p <0.005, ***p <0.0005; ****p <0.0001. **(C)** Quantification of cell separation defect based on Van-FL staining in Fig.6B. Unpaired t-test with Welch’s correction was performed for statistical analysis in panel B and C: *p <0.05; **p <0.005; ***p <0.0005; ****p <0.0001. **(D)** Anti-mCherry immunoblot analysis of whole cell culture. mCherry-SpsB, theoretical MW: 48.8 kD; mCherry-SpsB_∆2-27_, theoretical MW: 45.8 kD. The star indicates intact fusion protein. Circle indicates degradation products. The αSrtA blot serves as a loading control. The protein ladders in kDa are noted on the left side.


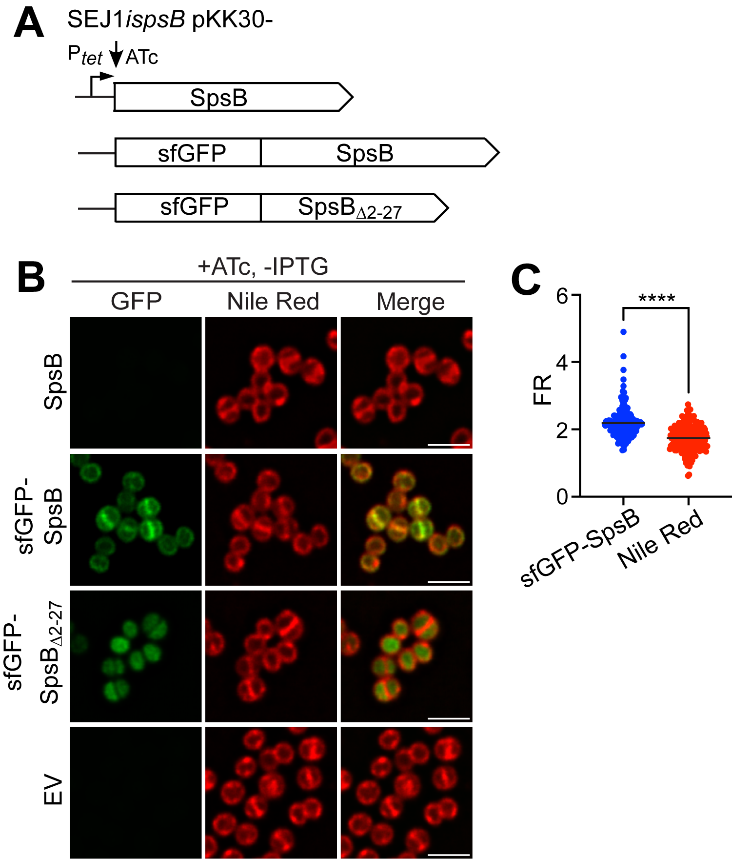


**Fig. S5.** SpsB is enriched at the septum of dividing staphylococcal cells. **(A)** Illustration of sfGFP-SpsB constructs. SpsB alone, sfGFP fused with SpsB or SpsB lacking its transmembrane domain (SpsB_∆2-27_) were cloned in pKK30itet vector expressed in SEJ1i*spsB*. **(B)** Fluorescence microscopy images showing the localization of sfGFP-SpsB and sfGFP-SpsB_Δ2-27_ in *spsB*-depleted cells (-IPTG). EV, pKK30itet empty vector control. Nile red (red) stains cell membrane. **(C)** Quantification of fluorescence intensity ratio (FR) of septum versus cell periphery. Unpaired t-test with Welch’s correction was performed for statistical analysis (****p <0.0001). Representative images and quantification are from three independent experiments.

**Table S1. Strains and plasmids used in this study.**

| **Strains or plasmids** | **Description^a^** | **Reference or source** |
| --- | --- | --- |
| *E. coli* |  |  |
| DC10B | Cloning strain | (1) |
| *S. aureus* |  |  |
| SEJ1 | RN4220∆*spa* (WYL112) | (2) |
| WYL745 | SEJ1 pCL*itet*-*sp_spa_-spa*,* Chl^R^, ATc-inducible | (3) |
| WYL480 | SEJ1 pCL*itet*, Chl^R^, ATc-inducible | (3) |
| WYL899 | SEJ1*srtA*::φΝΣ pCL*itet*-*sp_spa_*-*spa*,* Ery^R^, Chl^R^, ATc-inducible | (3) |
| WYL932 | SEJ1 pCL*itet-sp_spa_A37P_-spa*,* Chl^R^, ATc-inducible | This study |
| WYL1500 | SEJ1*ΔspsB::erm P_spac_-spsB,* pKK30*itet-mcherry-spsB,* Tmp^R^, Ery^R^, Chl^R^, ATc and IPTG-inducible | This study |
| WYL1520 | SEJ1 pKK30*itet,* Tmp^R^, ATc-inducible | This study |
| WYL1521 | SEJ1*ΔspsB::erm P_spac_-spsB,* pKK30*itet,* Tmp^R^, Ery^R^, Chl^R^, ATc and IPTG-inducible | This study |
| WYL931 | SEJ1*srtA*::φΝΣ pCL*itet*-*sp_spa_A37P_-spa**, Chl^R^, Ery^R^, ATc-inducible | This study |
| WYL895 | SEJ1*srtA*::φΝΣ , Ery^R^ | This study |
| WYL1146 | SEJ1 pKK30*itet*-*sp_spa_-spa*,* Chl^R^, Tmp^R^, ATc-inducible | This study |
| ANG2009 | SEJ1*ΔspsB::erm P_spac_-spsB,* Ery^R^, Chl^R^, IPTG-inducible (WYL657) | (4) |
| WYL1223 | SEJ1*ΔsrtA::-ΔspsB::erm* *P_spac_-spsB,* Chl^R^, Ery^R^, IPTG-inducible | This study |
| WYL1150 | SEJ1 pKK30*itet*-*sp_splE_-spa**, Chl^R^, Tmp^R^, ATc-inducible | This study |
| WYL1148 | SEJ1*srtA*::φΝΣ pKK30*itet*-*sp_spa-_spa*,* Chl^R^, Ery^R^, Tmp^R^, ATc-inducible | This study |
| WYL1152 | SEJ1*srtA*::φΝΣ pKK30*itet*-*sp_splE_-spa**, Chl^R^, Ery^R^, Tmp^R^, ATc-inducible | This study |
| WYL1226 | SEJ1*ΔsrtA*::-*ΔspsB::erm P_spac_-spsB,* pKK30*itet*-*sp_spa-_spa*,* Chl^R^, Ery^R^, Tmp^R^, ATc and IPTG-inducible | This study |
| WYL1229 | SEJ1*ΔsrtA*::-*ΔspsB::erm P_spac_-spsB,* pKK30*itet*-*sp_splE_-spa**, Chl^R^, Ery^R^, Tmp^R^, ATc and IPTG-inducible | This study |
| WYL1434 | SEJ1*ΔspsB::erm P_spac_-spsB,* pKK30*itet-sp_spa-_spa*,* Chl^R^, Ery^R^, Tmp^R^, ATc and IPTG-inducible | This study |
| WYL1435 | SEJ1*ΔspsB::erm P_spac_-spsB,* pKK30*itet*-*sp_splE_-spa**, Chl^R^, Ery^R^, Tmp^R^, ATc and IPTG-inducible | This study |
| WYL1554 | SEJ1*ΔspsB::erm P_spac-_spsB,* pKK30*itet-mcherry-**spsB_Δ2-27_,* Tmp^R^, Ery^R^, Chl^R^, ATc and IPTG-inducible | This study |
| WYL1553 | SEJ1*ΔspsB::erm P_spac_-spsB,* pKK30*itet-spsB,* Tmp^R^, Ery^R^, Chl^R^, ATc and IPTG-inducible | This study |
| WYL1557 | SEJ1*ΔspsB::erm P_spac_-spsB,* pKK30*itet-sfGFP-spsB,* Tmp^R^, Ery^R^, Chl^R^, ATc and IPTG-inducible | This study |
| WYL1558 | SEJ1*ΔspsB::erm P_spac_-spsB,* pKK30*itet-sfGFP -spsB_Δ2-27_,* Tmp^R^, Ery^R^, Chl^R^, ATc and IPTG-inducible | This study |
| WYL1616 | SEJ1, pKK30*itet-gfp_P7_-ltaS_WT_,*  Tmp^R^, Ery^R^, ATc-inducible | This study |
| WYL1617 | SEJ1, pKK30*itet-gfp_P7_-ltaS_S218P_,* Tmp^R^, Ery^R^, ATc-inducible | This study |
| WYL1521 | SEJ1*ΔspsB::erm P_spac_-spsB,* pKK30*itet,* Tmp^R^, Ery^R^, Chl^R^, ATc and IPTG-inducible | This study |
| WYL1613 | SEJ1*ΔspsB::erm P_spac_-spsB,* pKK30*itet-gfp_P7_-ltaS_WT_,* Tmp^R^, Ery^R^, Chl^R^, ATc and IPTG-inducible | This study |
| WYL1658 | SEJ1*ΔltaS::erm P_spac_-ltaS,*  pKK30*itet-gfp_P7_-ltaS_S218P_,* Tmp^R^, Ery^R^, ATc and IPTG-inducible | This study |
| ANG1786 | SEJ1*ΔltaS* suppressor 4S5 (WYL499) | (5) |
| **Plasmids** |  |  |
| pCL*itet* | pCL55 containing ATc-inducible P*_tet_* promoter, Amp^R^ (*E. coli*), Chl^R^ (*S. aureus*), ATc-inducible | (6) |
| pCL*itet*-*sp_spa_-spa** | LysM domain (413-457 aa of SpA) deletion in pCL*itet-sp_spa_*-*spa*, Amp^R^ (*E. coli*), Chl^R^ (*S. aureus*), ATc-inducible | (3) |
| pCL*itet-sp_spa_A37P_*-*spa** | A37P variant of pCL*itet-sp_spa_*-*spa**, Amp^R^ (*E. coli*), Chl^R^ (*S. aureus*), ATc-inducible | This study |
| pKK30*itet* | pKK30 containing ATc-inducible P*_tet_* promoter, Tmp^R^ (*E. coli*), Tmp^R^ (*S. aureus*), ATc-inducible | This study |
| pKK30*itet-mcherry*-*spsB* | *mcherry-spsB* fusion cloned in pKK30*itet*, Tmp^R^ (*E. coli*), Tmp^R^ (*S. aureus*), ATc-inducible | This study |
| pKK30*itet-sp_spa_-spa** | LysM domain (413-457 aa of SpA) deletion in pKK30*itet-sp_spa_-spa*, Tmp^R^ (*E. coli*), Tmp^R^ (*S. aureus*), ATc-inducible | This study |
| pKK30*itet-sp_splE_-spa** | SpA signal peptide replaced by SplE signal peptide, Tmp^R^ (*E. coli*), Tmp^R^ (*S. aureus*), ATc-inducible | This study |
| pKK30*itet-mcherry*-*spsB_Δ2-27_* | Transmembrane domain (2-27 aa of SpsB) deletion in pKK30*itet-mcherry*-*spsB*, Tmp^R^ (*E. coli*), Tmp^R^ (*S. aureus*), ATc-inducible | This study |
| pKK30*itet-spsB* | Full-length *spsB* cloned to pKK30*itet*, Tmp^R^ (*E. coli*), Tmp^R^ (*S. aureus*), ATc-inducible | This study |
| pKK30*itet-sfgfp*-*spsB* | *sfgfp-spsB* fusion cloned in pKK30*itet*, Tmp^R^ (*E. coli*), Tmp^R^ (*S. aureus*), ATc-inducible | This study |
| pKK30*itet-sfgfp*-*spsB_Δ2-27_* | Transmembrane domain (2-27 aa of SpsB) deletion in pKK30*itet-sfgfp*-*spsB*, Tmp^R^ (*E. coli*), Tmp^R^ (*S. aureus*), ATc-inducible | This study |
| pKK30*itet-gfp_p7_*-*ltaS_WT_* | *gfp_p7_-ltaS_WT_* cloned to pKK30*itet*, Tmp^R^ (*E. coli*), Tmp^R^ (*S. aureus*), ATc-inducible | This study |
| pKK30*itet-gfp_p7_*-*ltaS_S218P_* | *gfp_p7_-ltaS_S218P_* cloned to pKK30*itet*, Tmp^R^ (*E. coli*), Tmp^R^ (*S. aureus*), ATc-inducible | This study |

^a.^Abbreviations: Chl, chloramphenicol; Ery, erythromycin; Kan, kanamycin; Amp, ampicillin; Tmp, trimethoprim; ATc, anhydrotetracycline; IPTG, Isopropyl β-d-1-thiogalactopyranoside.

**Table S2. Primers used in this study.**

| **Primer No.** | | **Sequence** | **Description** |
| --- | --- | --- | --- |
| 416 SpA-A37P_F | tgcaaatgctccacaacacgatgaagctca | | pCL*itet-sp_spa_A37P_*-*spa** |
| 417 SpA-A37P_R | tcgtgttgtggagcatttgcagcaggt | | pCL*itet-sp_spa_A37P_*-*spa** |
| 562 SplE_F | acatacagggggtattaatatgaataaaaatataatcatcaaaagt | | pCL*itet*-*sp_splE_*-*spa** |
| 563 SplE_R | tgagcttcatcgtgttgcgcagctttagccgtttgttgaataccct | | pCL*itet*-*sp_splE_*-*spa** |
| 681 Pitet_NotI_F | aaagcggccgctggttaccgtgaagttaccatca | | pKK30*itet*-*sp_spa_*-*spa**,  pKK30*itet*-*sp_splE_-spa** |
| 682 SpA-SacI_R | aaagagctcccgcggttatagttcgcgacga | | pKK30*itet*-*sp_spa_-spa*,*  pKK30*itet*-*sp_splE_-spa** |
| 948 spsB_F | agggttcaGCTAGCgcaGGAatgaaaaaagaaatattggaatgga | | pKK30*itet-mcherry*-*spsB* |
| 887 spsB_R | atccccccgcgggtttaaacagatctctattaatttttagtattttcagga | | pKK30*itet-mcherry*-*spsB* |
| 886 gpmch_F | atatcaaatgacctaggaggttgtcgacatggtgagcaagggcgagga | | pKK30*itet-mcherry*-*spsB* |
| 837 gpmch_R | TCCtgcGCTAGCtgaacccttgtacagctcgtccatg | | pKK30*itet-mcherry*-*spsB* |
| 925 Pitet_F | tgttaatcactttacttttatctaat | | pKK30*itet* |
| 926 Pitet_R | tgattcggatccccgggtaccgAGCTCccgcgggtttaaacagatct | | pKK30*itet* |
| 972 spsB_F | tcggaggcatatcaaatgacctaggaggttgtcgacATGaaaaaagaaatattggaatgga | | pKK30*itet-spsB* |
| 973 spsB_R | accgAGCTCccgcgggtttaaacagatctctattaatttttagtattttcagga | | pKK30*itet-mcherry*-*spsB_Δ2-27_,*  pKK30*itet-spsB* |
| 974 mCh-spsB (lack 2-27)_F | acgagctgtacaagggttcaGCTAGCgcaGGAATGacgccatatacaattaaaggtga | | pKK30*itet-mcherry*-*spsB_Δ2-27_* |
| 641 pbp4_F | tgacctaggaggttgtcgacatgaaaaatttaatatctattatca | | pKK30*itet-sfgfp*-*spsB_Δ2-27_,*  pKK30*itet-sfgfp-spsB* |
| 642 pbp4_R | tgcGCTAGCtgcggcgcctccttttctttttctaaataaacgattga | | pKK30*itet-sfgfp*-*spsB_Δ2-27_,*  pKK30*itet-sfgfp-spsB* |
| 643 sfGFP_F | aggcgccgcaGCTAGCgcatcaaaaggtgaagaattatttaca | | pKK30*itet-sfgfp*-*spsB_Δ2-27_,*  pKK30*itet-sfgfp-spsB* |
| 644 sfGFP_R | aacagatctttatttatataattcatccatacca | | pKK30*itet-sfgfp*-*spsB_Δ2-27_,*  pKK30*itet-sfgfp-spsB* |
| 645 sfGFP_F | gttgtcgacatggcatcaaaaggtgaagaattatttaca | | pKK30*itet-sfgfp*-*spsB_Δ2-27_,*  pKK30*itet-sfgfp-spsB* |
| 646 sfGFP_R | GCTAGCtgcggcgcctcctttatataattcatccataccatgt | | pKK30*itet-sfgfp*-*spsB_Δ2-27_,*  pKK30*itet-sfgfp-spsB* |
| 994 sfGFP_F | tcggaggcatatcaaatgacctaggaggttgtcgacatggcatca | | pKK30*itet-sfgfp*-*spsB_Δ2-27_,*  pKK30*itet-sfgfp-spsB* |
| 995 sfGFP_R | tcttttttCATTCCtgcGCTAGCtgcggcgcctcctttatataattca | | pKK30*itet-sfgfp*-*spsB_Δ2-27_,*  pKK30*itet-sfgfp-spsB* |
| 673 srtA_1F | GGGGACAAGTTTGTACAAAAAAGCAGGCTacgaaaatgcgcttgtaacaagct | | *ΔsrtA* |
| 674 srtA_2R | agcgtaatagattaacgttaaggctccttttatacatttca | | *ΔsrtA* |
| 675 srtA_3F | aggagccttaacgttaatctattacgctaatggatgaata | | *ΔsrtA* |
| 676 srtA_4R | GGGGACCACTTTGTACAAGAAAGCTGGGTacacataatttatccgatttaagtgct | | *ΔsrtA* |
| 677 srtA_5F | acgtcgcaaaccctaagacact | | *ΔsrtA* confirmation |
| 678 srtA_6R | agcattgtatattggattggttcagt | | *ΔsrtA* confirmation |
| 679 srtA_7F | tcgctcagcatgattatcgttttca | | *ΔsrtA* confirmation |
| 680 srtA_8R | agatgaagttacaaacgctttagaca | | *ΔsrtA* confirmation |
| 1023 LtaS_F | agcgctagcaagcgaagatgacttaacaaaagt | | pKK30*itet*-*gfp_P7_*-*ltaS_WT_* |
| 1024 LtaS_R | tcatcttcgcttgctagcgctttttgttga | | pKK30*itet*-*gfp_P7_*-*ltaS_WT_* |

**References**

1. Monk IR, Shah IM, Xu M, Tan MW, Foster TJ. 2012. Transforming the untransformable: application of direct transformation to manipulate genetically *Staphylococcus aureus* and *Staphylococcus epidermidis*. MBio 3.

2. Gründling A, Schneewind O. 2007. Genes required for glycolipid synthesis and lipoteichoic acid anchoring in *Staphylococcus aureus*. J Bacteriol 189:2521-30.

3. Zhang R, Shebes MA, Kho K, Scaffidi SJ, Meredith TC, Yu W. 2021. Spatial regulation of protein A in Staphylococcus aureus. Mol Microbiol 116:589-605.

4. Wormann ME, Reichmann NT, Malone CL, Horswill AR, Grundling A. 2011. Proteolytic cleavage inactivates the Staphylococcus aureus lipoteichoic acid synthase. J Bacteriol 193:5279-91.

5. Corrigan RM, Abbott JC, Burhenne H, Kaever V, Grundling A. 2011. c-di-AMP is a new second messenger in Staphylococcus aureus with a role in controlling cell size and envelope stress. PLoS Pathog 7:e1002217.

6. Gründling A, Schneewind O. 2007. Synthesis of glycerol phosphate lipoteichoic acid in *Staphylococcus aureus*. Proc Natl Acad Sci U S A 104:8478-83.
